# Tricuspid annular plane systolic excursion by cardiac MRI has poor correlation with RVEF in pediatric patients

**DOI:** 10.1186/1532-429X-15-S1-O40

**Published:** 2013-01-30

**Authors:** Emem Usoro, Jonathan H Soslow, David Parra

**Affiliations:** 1 Pediatrics, Division of Pediatric Cardiology, Vanderbilt University Medical Center, Nashville, TN, USA; 2 Meharry Medical College, Nashville, TN, USA

## Background

Cardiovascular MRI (CMR) assessment of ventricular function in patients with RV pathology requires contouring the right ventricle, which can be time-consuming due to its abnormal shape and extensive trabeculations. A semi-quantitative method to assess RV function in this patient population would be beneficial and would allow for rapid reporting of results. A recent study demonstrated a high correlation of tricuspid annular plane systolic excursion as measured by CMR (MR-TAPSE) with RVEF in adult patients. In this study, we evaluated whether MR-TAPSE is a feasible alternative to assess RV function in children and young adults with a broad range of body surface areas (BSA).

## Methods

A retrospective review from 2007-2012 identified: 49 patients with normal cardiac anatomy aged 18 years or under who underwent CMR (group 1), and 57 patients with Tetralogy of Fallot status post repair aged 18 years or under (group 2). Measurements of MR-TAPSE in group 1 were performed by three reviewers blinded to outcome. MR-TAPSE was measured in the four-chamber view of the cine MRI images. Agreement among reviewers was assessed using intraclass correlation coefficient (ICC). Linear regression analysis was used to assess MR-TAPSE prediction of RVEF.

## Results

**Figure 1:**
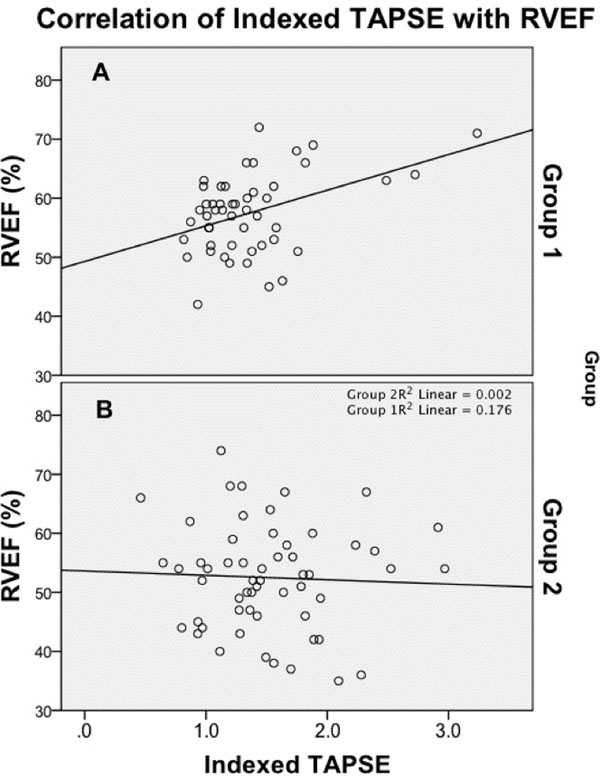
Indexed tricuspid annular plane systolic excursion by CMR (MR-TAPSE) had only moderate correlation with RVEF in group 1, or children and adolescents with normal CMR (A). Indexed MR-TAPSE had poor correlation with RVEF in group 2, or children and adolescents with Tetralogy of Fallot (B).

The mean age of patients was 14.9 ± 3.3 years (range 2.7-18.8 years) in group 1 and 13.5 ± 3.4 years (range 4.3-18.9 years) in group 2. The mean BSA was 1.8 ± 0.4m2 (range 0.5-2.7m2) in group 1 and 1.4 ± 0.4m2 (range 0.7-2.3m2) in group 2. ICC demonstrated excellent correlation for individual TAPSE measures, suggesting low inter-observer variability (ICC 0.872 for diastole and ICC 0.784 for systole). The average MR-TAPSE in group 1 had only moderate correlation with RVEF (r=0.3, p = 0.036) and only mild improvement in correlation after normalization with BSA (r=0.42, p<0.05) (Figure 1A). RVEF in group 2 did not correlate with either MR-TAPSE (r=0.027, p=0.844) or indexed MR-TAPSE (r=0.043, p=0.75) (Figure 1B).

## Conclusions

Although MR-TAPSE has been found to be a reliable method of semi-quantification of RV function in adults, its use in the pediatric population may not be suitable, despite the fact that CMR provides better resolution and more accurate delineation of the cardiac apex than 2-dimensional echocardiography or M-mode. This may be partially due to the significant variability in patient size; in group 2, the poor correlation is also likely related to RV dilatation and aneurysmal RVOT.

## Funding

None.

